# The Novel Arylamidine T-2307 Selectively Disrupts Yeast Mitochondrial Function by Inhibiting Respiratory Chain Complexes

**DOI:** 10.1128/AAC.00374-19

**Published:** 2019-07-25

**Authors:** Kohei Yamashita, Taiga Miyazaki, Yoshiko Fukuda, Junichi Mitsuyama, Tomomi Saijo, Shintaro Shimamura, Kazuko Yamamoto, Yoshifumi Imamura, Koichi Izumikawa, Katsunori Yanagihara, Shigeru Kohno, Hiroshi Mukae

**Affiliations:** a Department of Pharmacology Research (Toyama Works), Pharmaceutical and Healthcare Research Laboratories, FUJIFILM Corporation, Toyama, Japan; b Department of Respiratory Medicine, Nagasaki University Graduate School of Biomedical Sciences, Nagasaki, Japan; c Department of Respiratory Medicine, Nagasaki University Hospital, Nagasaki, Japan; d Department of Infectious Diseases, Nagasaki University Graduate School of Biomedical Sciences, Nagasaki, Japan; e Department of Sales Promotion, FUJIFILM Toyama Chemical Co., Ltd., Tokyo, Japan; f Development Division, FUJIFILM Toyama Chemical Co., Ltd., Tokyo, Japan; g Department of Laboratory Medicine, Nagasaki University Graduate School of Biomedical Sciences, Nagasaki, Japan

**Keywords:** *Candida albicans*, antifungal, mechanism of action, respiratory chain, yeast

## Abstract

The novel arylamidine T-2307 exhibits broad-spectrum *in vitro* and *in vivo* antifungal activities against clinically significant pathogens. Previous studies have shown that T-2307 accumulates in yeast cells via a specific polyamine transporter and disrupts yeast mitochondrial membrane potential. Further, it has little effect on rat liver mitochondrial function. The mechanism by which T-2307 disrupts yeast mitochondrial function is poorly understood, and its elucidation may provide important information for developing novel antifungal agents. This study aimed to determine how T-2307 promotes yeast mitochondrial dysfunction and to investigate the selectivity of this mechanism between fungi and mammals. T-2307 inhibited the respiration of yeast whole cells and isolated yeast mitochondria in a dose-dependent manner. The similarity of the effects of T-2307 and respiratory chain inhibitors on mitochondrial respiration prompted us to investigate the effect of T-2307 on mitochondrial respiratory chain complexes. T-2307 particularly inhibited respiratory chain complexes III and IV not only in Saccharomyces cerevisiae but also in Candida albicans, indicating that T-2307 acts against pathogenic fungi in a manner similar to that of yeast. Conversely, T-2307 showed little effect on bovine respiratory chain complexes. Additionally, we demonstrated that the inhibition of respiratory chain complexes by T-2307 resulted in a decrease in the intracellular ATP levels in yeast cells. These results indicate that inhibition of respiratory chain complexes III and IV is a key factor for selective disruption of yeast mitochondrial function and antifungal activity.

## Abstract (precis)

The novel arylamidine T-2307 exhibits broad-spectrum *in vitro* and *in vivo* antifungal activities against clinically significant pathogens. Previous studies have shown that T-2307 accumulates in yeast cells via a specific polyamine transporter and disrupts yeast mitochondrial membrane potential. Further, it has little effect on rat liver mitochondrial function.

## INTRODUCTION

Invasive mycoses are life-threatening infections particularly affecting immunocompromised individuals because of aggressive therapies (e.g., anticancer chemotherapy or organ transplant) or individuals with immunosuppressive infections, such as HIV/AIDS (1). For the treatment of invasive mycoses, three main classes of antifungal drugs are used: azoles, echinocandins, and polyenes. Despite these treatment options, several studies have shown the emergence of resistant organisms such as azole-resistant *Candida* spp., echinocandin-resistant *Candida* spp., or intrinsically multidrug-resistant Candida auris (2–6). It is difficult to treat mycoses caused by these resistant fungi because of limited treatment options; therefore, there is a critical need for a new class of drugs functioning with novel modes of action.

**FIG 1:**
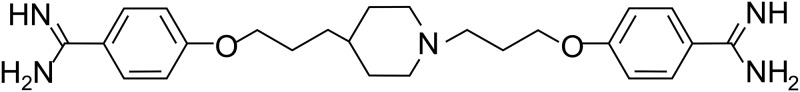
Chemical structure of T-2307.

T-2307 (Fig. 1), having a novel chemical structure, exhibits *in vitro* broad-spectrum activity and good therapeutic effects in murine infection models against fungal pathogens, including *Candida* spp., *Cryptococcus* spp., and Aspergillus fumigatus (7–9). Furthermore, T-2307 presents potent antifungal *in vitro* and *in vivo* activities against echinocandin-resistant *Candida* spp. and azole-resistant Candida albicans (7, 10, 11). The activities of T-2307 against these resistant strains are attributable to its unique mode of action.

The mechanism of action of T-2307 has been investigated in several studies (12–14). T-2307 is incorporated into yeast cells by a specific polyamine transporter related to the uptake of spermine and spermidine, and its concentration reportedly increases >3,000-fold in C. albicans from the extracellular medium (12, 13). Once inside the cytosol, T-2307 reaches mitochondria and disrupts the mitochondrial membrane potential (MMP), resulting in mitochondrial dysfunction (14). Remarkably, T-2307 has little effect on rat liver mitochondrial functions, indicating that T-2307 is a selective inhibitor of yeast mitochondrial function (14). Therefore, elucidating the mechanism for the action of T-2307 on mitochondria, which is currently poorly understood, will facilitate the development of a novel antifungal drug.

This study aimed to thoroughly understand the mechanism by which T-2307 contributes to yeast mitochondrial dysfunction and antifungal activity. The data obtained in this study indicate that inhibition of the respiratory chain system is critical for disrupting mitochondrial function and exhibiting antifungal activity. In addition, we compared the levels of selectivity of the effect on respiratory chain systems between fungi and mammals.

## RESULTS

### Effects of T-2307 on yeast respiration in whole yeast cells and yeast mitochondria.

**FIG 2:**
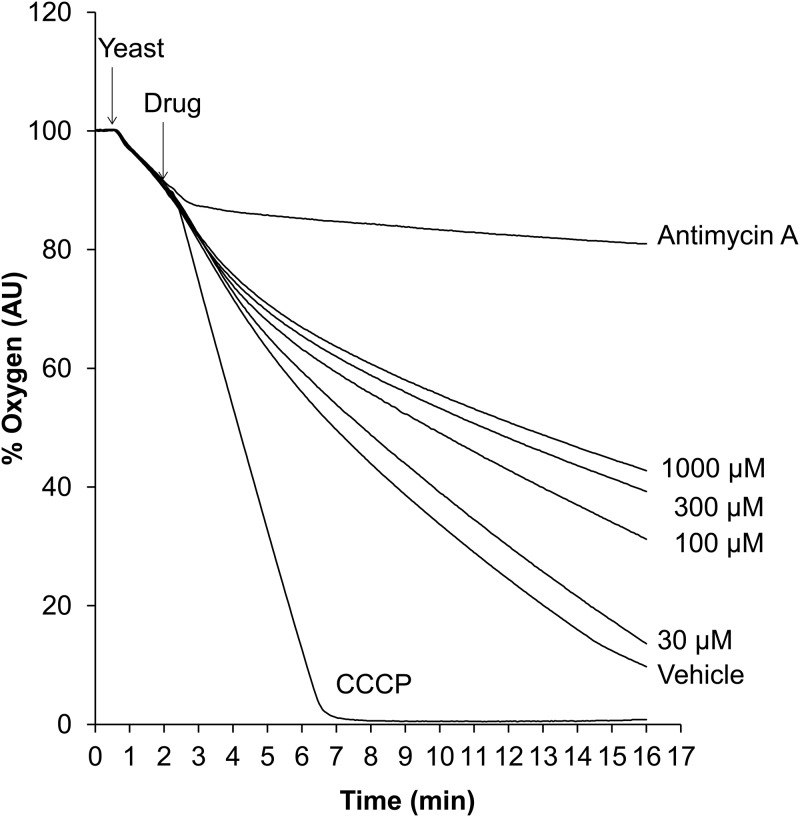
Dose-dependent effect of T-2307 on oxygen consumption in whole yeast cells. Oxygen consumption was measured in distilled water at a cell density of 3 × 10^7^ cells/ml. Antimycin A and CCCP were added at final concentrations of 10 μg/ml and 4 μM, respectively. T-2307 was added at final concentrations of 30, 100, 300, and 1,000 μM. Distilled water was added to the control group as a vehicle control. The time of yeast cell and agent addition is indicated by the arrows. All measurements were conducted at 23°C. Curves are representative of three independent experiments. AU, arbitrary units.

Analyzing mitochondrial respiration is an informative way of assessing mitochondrial function. To investigate the effect of T-2307 on yeast respiration, we measured oxygen consumption in the presence of T-2307, antimycin A, and carbonyl cyanide m-chlorophenylhydrazone (CCCP) using whole yeast cells. As shown in Fig. 2, CCCP treatment stimulated oxygen consumption, whereas antimycin A treatment completely and immediately inhibited respiration; thus, it can be inferred that CCCP and antimycin A served as positive controls with respect to uncoupling and inhibition of mitochondrial respiration, respectively. T-2307 treatment caused dose-dependent inhibition of respiration. However, stimulated respiration was not observed upon T-2307 treatment at any concentration. Overall, these results suggested that T-2307 inhibits yeast mitochondrial respiration.

**FIG 3:**
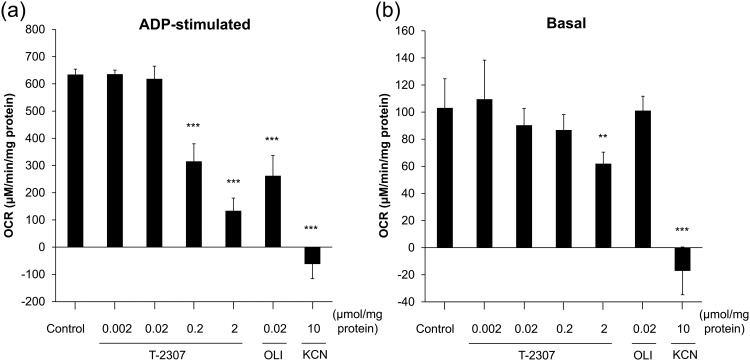
Effect of T-2307 on oxygen consumption in isolated yeast mitochondria. Oxygen consumption of isolated mitochondria was measured using a MitoXpress probe. The inhibitory effects of T-2307, oligomycin A (OLI), and KCN were evaluated in the presence of ADP (ADP-stimulated condition) (a) and in the absence of ADP (basal condition) (b). Oxygen consumption rates (OCRs) were determined from the slope of the initial 7.5 min of the time-oxygen concentration curve. The values represent the mean and standard deviation values of pooled data from two independent experiments, each performed in triplicate (total six replicates per group). Statistical analysis was performed using the parametric Dunnett’s multiple-comparison test. Asterisks indicate a significant difference from the control (**, *P < *0.01; ***, *P < *0.001).

Next, we investigated the effect of T-2307 on mitochondrial respiration using intact mitochondria. Respiratory control ratio (RCR) values for prepared mitochondria were found to be a minimum of 4.8 in all experiments, indicating that the prepared mitochondria were well coupled (14, 15). To identify whether T-2307 inhibits the respiratory chain or ATP synthase, we measured mitochondrial oxygen consumption in the presence or absence of ADP. As shown in Fig. 3, T-2307 causes a dose-dependent decrease in the mitochondrial oxygen consumption rate (OCR) under ADP-stimulated (in the presence of ADP) and basal (in the absence of ADP) conditions. Oligomycin A, an ATP synthase inhibitor, did not decrease the OCR under basal conditions at the same concentration that significantly inhibited ADP-stimulated respiration. On the other hand, potassium cyanide (KCN), a respiratory chain inhibitor, completely inhibited respiration under both basal and ADP-stimulated conditions. Notably, the inhibition patterns of oligomycin A and KCN were consistent with those reported previously (16). The inhibition pattern of T-2307 was similar to that of respiratory chain inhibitor. These results indicate that T-2307 inhibits the yeast mitochondrial respiratory chain.

### Enzyme inhibitory activity of T-2307 against yeast respiratory chain complexes.

In eukaryotes, the mitochondrial respiratory chain comprises four enzymatic complexes (complexes I to IV) embedded in the inner mitochondrial membrane (17), whereas the Saccharomyces cerevisiae respiratory chain lacks complex I (18). We investigated the effect of T-2307 on the enzymatic activity of each complex in yeast. T-2307 exhibits potent antifungal activity not only against S. cerevisiae but also against pathogenic fungi, including against C. albicans (7). Therefore, we also investigated whether T-2307 inhibits C. albicans mitochondrial respiratory chain complexes.

**FIG 4:**
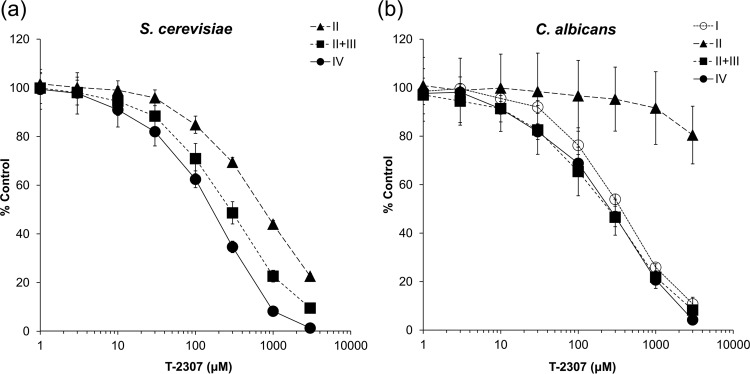
Dose-response curves representing the effect of T-2307 on the enzymatic activities of respiratory chain complexes in Saccharomyces cerevisiae (a) and Candida albicans (b). The reaction rate of each enzyme was determined by calculating the slope of the linear portion of the curve representing the trace of the reaction of each complex. Percent control was calculated based on the slope in the control. The values represent the mean and standard deviation values of pooled data from two independent experiments, each performed in triplicate (total six replicates per group).

The percent inhibition values of positive controls are shown in Table S1 in the supplemental material. These results were consistent with those reported previously (17, 19, 20). As shown in Fig. 4, T-2307 inhibited all complexes in a dose-dependent manner in both species except complex II of C. albicans. The 50% inhibitory concentration (IC_50_) values of T-2307 for complexes II plus III and IV were lower than those for the other complexes in both species (Table 1). Although the IC_50_ value of T-2307 for complex I was similar to that for complexes II plus III and IV in C. albicans, the IC_50_ value of T-2307 against NADH dehydrogenase activity which involves complex I activity was 1,999 μM. It was reported that rotenone partially inhibits NADH dehydrogenase activity and respiration of isolated mitochondria from C. albicans because of the presence of rotenone-insensitive NADH dehydrogenase (19, 20). Thus, rotenone shows a low level of inhibition of cell proliferation (20). Because the IC_50_ value of T-2307 for NADH dehydrogenase was much higher than that for complex I, T-2307 was suggested to not have an effect on rotenone-insensitive NADH dehydrogenase. Therefore, the inhibition of complex I in C. albicans was considered to not contribute to the mitochondrial dysfunction and antifungal activity of T-2307. The IC_50_ values against respiratory chain complex II were much higher than those against complexes II plus III, indicating that T-2307 inhibited the enzymatic activity of complex III more strongly than that of complex II in both species (Table 1). Therefore, these results suggest that T-2307 induces mitochondrial dysfunction mainly via inhibition of complexes III and IV in both species.

**TABLE 1:** The 50% inhibitory concentration values of T-2307 against respiratory chain complexes in Saccharomyces cerevisiae, Candida albicans, and bovine heart mitochondria^*a*^

Source	IC_50_ (μM) ± SD		
	S. cerevisiae	C. albicans	Bovine heart
Complex I	Nonexistence	344 ± 32	>3,000
Complex II	755 ± 73	>3,000	Not determined
Complex II + III	271 ± 37	220 ± 55	>3,000
Complex IV	149 ± 23	225 ± 23	>3,000

a The values represent means and standard deviations of pooled data from two independent experiments, each performed in triplicate in S. cerevisiae and C. albicans mitochondria and 6 to 8 times in bovine heart mitochondria.

### Effects of T-2307 on mammalian mitochondrial respiratory chain complexes.

We previously reported that T-2307 has little effect on mammalian MMP and respiration (14). Therefore, we hypothesized that the levels of enzyme inhibitory activity of T-2307 differ between yeast and mammalian cells. Thus, we investigated the effect of T-2307 on the enzymatic activity of each complex in mammalian cells using mitochondria isolated from bovine heart.

The percent inhibition values of positive controls are shown in Table S1. These results were consistent with those reported previously (17) and with the data described in the manufacturer’s instructions. The IC_50_ values for T-2307 were >3,000 μM for all complexes (Table 1). At the highest concentration (3,000 μM) of T-2307, the enzymatic activities of complex I, complexes II plus III, and complex IV remained 68%, 93%, and 92%, respectively. The IC_50_ values for complexes II plus III and complex IV in bovine heart mitochondria were >10-fold higher than those in yeast mitochondria. These results suggest that the differences between mammalian and yeast cells in terms of the inhibitory effect of T-2307 on respiratory chain complexes contribute to the selective inhibition of yeast mitochondrial function.

### Intracellular ATP levels in yeast.

**FIG 5:**
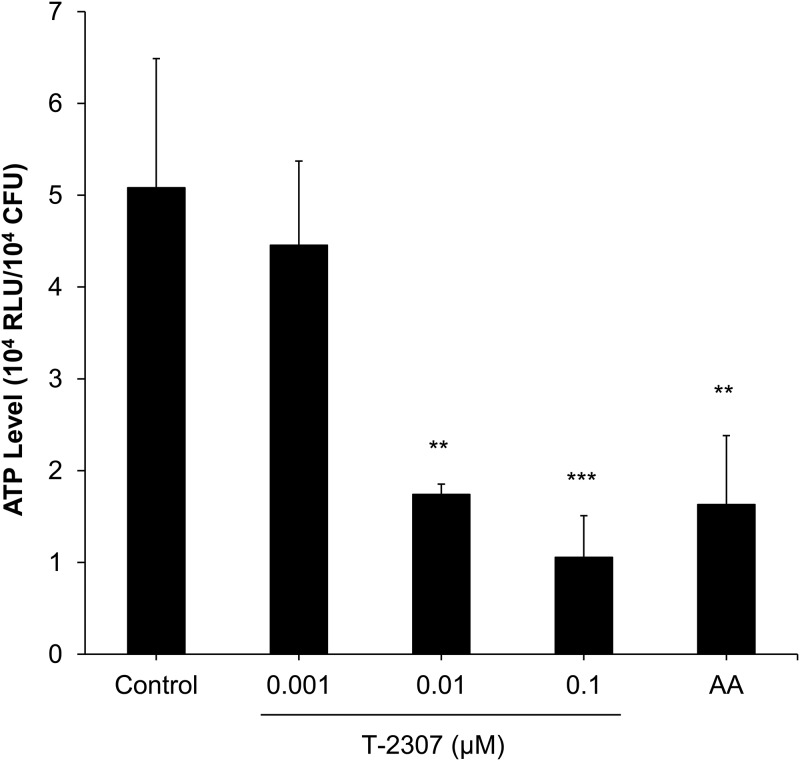
Effect of T-2307 on intracellular ATP levels in Saccharomyces cerevisiae. Cells were incubated for 8 h at 30°C in a semisynthetic medium in the presence or absence of T-2307 and antimycin A (AA). AA at 0.5 μg/ml was used as a positive control. ATP levels were measured using a luminescence assay. Intracellular ATP levels were expressed as relative light units (RLU)/CFU. Data are expressed as mean and standard deviation values of results from three independent experiments. Statistical analysis was performed using the parametric Dunnett’s multiple-comparison test. Asterisks indicate a significant difference from the control (**, *P < *0.01; ***, *P < *0.001).

We hypothesized that T-2307 inhibits yeast growth by interfering with ATP production because the respiratory chain plays an important role in the production of ATP. After an 8-h exposure to control media or media containing T-2307 or the respiratory chain inhibitor antimycin A, intracellular ATP levels were measured. As expected, intracellular ATP level was significantly decreased by antimycin A treatment (Fig. 5). T-2307 treatment at a concentration of ≥0.01 μM significantly decreased intracellular ATP levels (Fig. 5). These results suggested that T-2307 suppressed ATP production through inhibition of the respiratory chain. Moreover, to investigate whether the ATP loss caused growth inhibition, we determined the MIC of T-2307 for yeast in a semisynthetic medium used to measure intracellular ATP levels. The MIC of T-2307 was 0.0068 μg/ml (0.0156 μM), corresponding to the concentration at which T-2307 caused a >50% reduction in intracellular ATP levels. Additionally, approximately an 80% reduction in intracellular ATP level and an >75% growth inhibition were observed at the concentration of 0.055 μg/ml (0.125 μM). These results suggest that T-2307 inhibits yeast growth by interfering with ATP production in a dose-dependent manner.

## DISCUSSION

**FIG 6:**
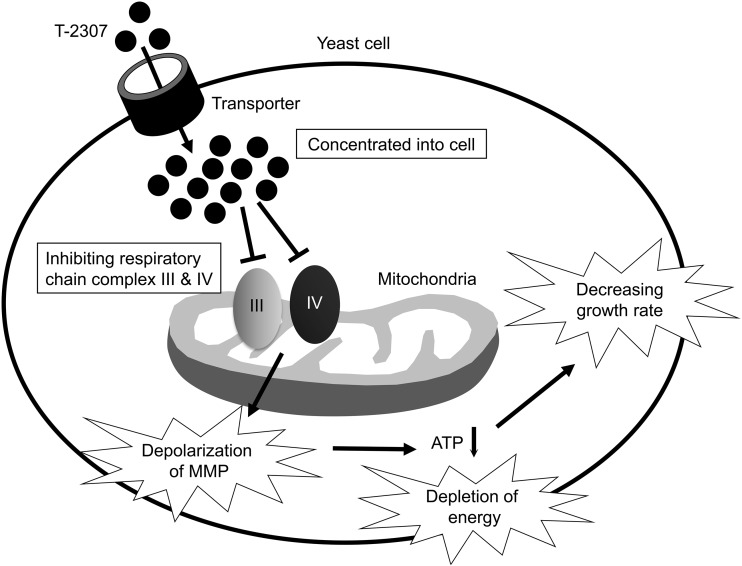
The hypothesized mechanism of action of T-2307. T-2307 is concentrated in yeast cells by a specific transporter (12, 13). T-2307 inhibits mitochondrial respiratory chain complexes, which leads to a decrease in intracellular ATP levels. Finally, cell growth decreases due to energy depletion.

In the current study, we revealed that T-2307 primarily inhibited respiratory chain complexes III and IV in yeast mitochondria. We propose that the interaction of T-2307 with these complexes is a key element of the antifungal activity of T-2307. The proposed growth-inhibitory mechanism of T-2307 is similar to that illustrated in Fig. 6. In addition, we revealed that T-2307 showed little effect on respiratory chain complexes from bovine heart mitochondria. These results indicate that T-2307 would be a selective inhibitor of yeast respiratory chain complexes.

A previous study showed that T-2307 disrupted MMP in whole yeast cells and in isolated mitochondria of S. cerevisiae and C. albicans (14). In the present study, investigation revealed that T-2307 suppressed mitochondrial respiration in whole yeast cells (Fig. 2) and in isolated mitochondria (Fig. 3). Moreover, we found that T-2307 specifically inhibited mitochondrial respiratory chain complexes III and IV in S. cerevisiae and C. albicans (Fig. 4; see also Table 1). It is known that the MMP collapses because of the presence of respiratory chain complex inhibitors, such as antimycin A or KCN (21–23). Therefore, the results of the present study suggested that the collapse of yeast MMP caused by the presence of T-2307 was a result of the inhibition of respiratory chain complexes. However, the reported concentrations of T-2307 causing 50% inhibition of MMP in the mitochondria isolated from S. cerevisiae and C. albicans were 5-fold to 20-fold lower than the IC_50_ values against complexes III and IV of these species (14). Nevertheless, we consider that inhibition of respiratory chain complexes is most likely a major mechanism of disruption of MMP because T-2307 showed highly selective inhibition against yeast respiratory chain complexes compared with mammalian cell respiratory chain complexes, which is consistent with the results of a study demonstrating that T-2307 showed selective inhibition against MMP of yeast mitochondria compared with MMP of mammalian cell mitochondria (14).

The IC_50_ values of T-2307 for complexes III and IV in S. cerevisiae and C. albicans (Table 1) are much higher than the MICs reported previously (0.002 μM for S. cerevisiae and 0.004 μM for C. albicans) (14). One possible explanation for this gap is that T-2307 accumulates in yeast cells as a consequence of active transport. We previously reported that T-2307 is actively transported into S. cerevisiae and C. albicans cells by a specific polyamine transporter related to the uptake of spermine and spermidine (12, 13). By the activity of this transporter, T-2307 is concentrated in C. albicans cells to concentrations of approximately >3,000-fold from the extracellular medium (12). In our experiments, T-2307 was also concentrated in S. cerevisiae cells to approximately 6,000-fold from the extracellular medium (data not shown). Therefore, we considered that the combination of inhibition of complexes and T-2307 accumulation in yeast cells contributed to the growth-inhibitory effect of T-2307 on yeast.

Both intracellular ATP levels and yeast growth were decreased by T-2307 in a dose-dependent manner within the same range of concentrations (Fig. 5). The mitochondrial respiratory chain complexes are known to play important roles in cellular ATP production (24). Reportedly, the antifungal action of respiratory chain inhibitors, such as antimycin A, UK-2A, and AS2077715, reduces ATP levels in fungi (25, 26). Therefore, it is suggested that the depletion of cellular ATP through the inhibition of respiratory chain complexes contributes to the growth-inhibitory effect of T-2307 on yeast.

Evaluation of the effect of T-2307 on bovine heart respiratory chain complexes revealed that the inhibitory effects of T-2307 on mammalian respiratory chain complexes are much weaker than those on yeast respiratory chain complexes (Table 1). These findings are consistent with those of a study showing that T-2307 had little effect on the MMP and on respiration in rat liver mitochondria (14). As almost all respiratory chain inhibitors bind to the catalytic center of complexes (27–29), it is reasonable to infer that T-2307 inhibits complexes III and IV via a similar mechanism. Although these targets are highly conserved among eukaryotes, the levels of sequence identity of catalytic core subunits between yeast and bovine animals are 51% to 58% for complex III and 43% to 59% for complex IV (30, 31). It is suggested here that these differences contribute to selective inhibition by a compound. For example, ilicicolin H is proposed to inhibit yeast respiratory chain complex III by interacting with amino acid residues that are different from those present in higher eukaryotes in complex III center N (28, 32). Thus, ilicicolin H showed >70-fold selectivity in comparisons between yeast and bovine complex III (32, 33). Therefore, it is possible that T-2307 interacts with yeast amino acid residues that are different from those found in mammalian complexes III and IV. Additional studies are required to elucidate the mechanism of T-2307-induced enzyme inactivation and selectivity mechanisms.

In conclusion, our results indicate that T-2307 inhibits yeast respiratory chain complexes III and IV. In addition, we revealed that T-2307 shows high selectivity in the inhibition of respiratory chain complexes in comparisons between fungal and mammalian cells. These findings indicate that respiratory chain complexes would be suitable targets of antifungal agents. Further study is required to elucidate the mechanism of selective inhibition of fungal respiratory chain complexes III and IV because it will provide essential information for understanding the selectivity of T-2307 and designing novel antifungal agents.

## MATERIALS AND METHODS

### Materials.

T-2307 was synthesized by FUJIFILM Toyama Chemical Co., Ltd. (Tokyo, Japan). Antimycin A, rotenone, 2-thenoyltrifluoroacetone (TTFA), and oligomycin A were purchased from Sigma-Aldrich (St. Louis, MO, USA). KCN and CCCP were purchased from FUJIFILM Wako Pure Chemical Corporation (Osaka, Japan). Malonic acid was purchased from Tokyo Chemical Industry (Tokyo, Japan).

### Strains.

The Saccharomyces cerevisiae and Candida albicans strains were obtained from the American Type Culture Collection [catalog no. 24657 (D273-10B) and MYA-2876 (SC5314)].

### Measurement of oxygen consumption of whole yeast cells.

Oxygen consumption of whole yeast cells was measured using an oxygen electrode installed in a closed cell (model 5300; YSI Incorporated) as described previously (34). S. cerevisiae was grown in yeast extract-peptone-glycerol (YPG) medium (1% yeast extract, 2% peptone, and 2% glycerol) for 16 h at 30°C with gentle shaking. The cells were harvested and suspended in distilled water (3 × 10^9^ cells/ml). Oxygen consumption was measured in distilled water at a cell density of 3 × 10^7^ cells/ml. All measurements were performed at 23°C.

### Preparation of yeast intact mitochondria.

S. cerevisiae from frozen stock (−80°C) was inoculated into yeast extract-peptone-glucose (YPD) medium (1% yeast extract, 2% peptone, and 2% glucose) and grown for 24 h at 30°C. The cultured cells were subcultured into yeast extract-peptone-galactose (YPGal) medium (1% yeast extract, 2% peptone, 2% galactose, and 0.1% glucose) and incubated overnight with continuous shaking at 30°C. When the optical density at 600 nm reached approximately 1.0, the cells were collected by centrifugation (3,000 × *g* at room temperature for 5 min). Intact mitochondria were isolated as per a previously described protocol (35) with minor modifications as follows. Yeast cells were converted to spheroplasts by incubation with Zymolyase-20T (Nacalai Tesque, Inc., Kyoto, Japan) (4 mg of Zymolyase-20T/g [wet weight] of cells) and disrupted using a Dounce homogenizer. The crude mitochondrial fraction was isolated by differential centrifugation (35). The final pellet of the crude mitochondrial fraction was suspended in respiration buffer (250 mM sucrose, 15 mM KCl, 1 mM EGTA, 5 mM MgCl_2_, and 30 mM K_2_HPO_4_, pH 7.4), which was used as a mitochondrial stock solution. The mitochondrial protein concentration was determined using the DC protein assay (Bio-Rad Laboratories, Inc., Hercules, CA, USA) following the manufacturer’s instructions. The mitochondrial stock solution was maintained on ice during the oxygen consumption experiment.

### Measurement of mitochondrial oxygen consumption.

The mitochondrial oxygen consumption was measured using a phosphorescent oxygen-sensitive probe, MitoXpress-Xtra (Agilent Technologies, Santa Clara, CA, USA), as described previously (16, 36). Succinate was used as a respiratory substrate in this assay. Evaluation was performed using only succinate (basal conditions) or both succinate and ADP (ADP-stimulated conditions). Briefly, the reconstituted MitoXpress probe solution (0.05 μM final concentration), mitochondrial solution, and stock solution of drugs were dispensed in each well of a flat-bottom 96-well black plate. Mitochondrial protein was added to each well; 40 μg and 80 μg of mitochondrial protein were used for the experiments performed under ADP-stimulated and basal conditions, respectively. Subsequently, succinate (25 mM final concentration) without or with ADP (1.65 mM final concentration) in the respiration buffer was added to each well. Finally, all wells were sealed with mineral oil, and measurements were immediately initiated. A microplate reader, PHERAstar Plus (BMG Labtech GmbH, Ortenberg, Germany), was used to read. The time-resolved fluorescence protocol was set to read the plate at 1.5-min intervals for 60 min at an excitation wavelength of 337 nm, an emission wavelength of 665 nm, a delay time of 40 μs, and a gate time of 100 μs at 30°C.

The mitochondrial oxygen consumption was calculated using the following transformation to convert fluorescence value to oxygen concentration for each sample at each time (16):

$${\text{[O}}_{2}](t\text{) = \{235}\times I\text{a}\times \text{[}I0-I\text{(}t\text{)]\}/[}I\text{(}t\text{) × (}I\text{0}-I\text{a)]}$$

At 30°C, the oxygen concentration in the well was 235 μM. *I*a represents the signal of the no-mitochondria control (O_2_ saturated). *I*0 represents the maximum signal (0% O_2_), which is the highest intensity seen under the ADP-stimulated condition. *I*(*t*) represents the signal of each sample at each time point (*t*). For quantitative assessment, OCR was determined from the slope of the initial 7.5 min. OCR is expressed as micromolar of O_2_ per minute per milligram of protein.

The drug concentration was expressed as micromoles per milligram of protein. Because the effect of drug on OCR changes with protein concentration (16, 36), the drug concentration was normalized to the mitochondrial protein concentration. With the unit of measurement changed to micromolar, 1 μmol/mg protein is equivalent to 200 μM under ADP-stimulated conditions and to 400 μM under basal conditions.

To evaluate the functionality of isolated mitochondria, RCR was calculated as previously described (36). The levels of oxygen consumption at 40 and 80 μg of mitochondria were measured under basal and ADP-stimulated conditions prior to drug testing and determined the ratio of OCR under ADP-stimulated condition to OCR under basal conditions from sample containing the same concentration of mitochondria, obtaining RCR.

### Assessment of respiratory chain enzymatic activities.

For assessing the mitochondrial respiratory chain enzymatic activities of S. cerevisiae and C. albicans, mitochondrial proteins were isolated using a Minute yeast mitochondria enrichment kit (Invent Biotechnologies Inc., Plymouth, MN, USA). S. cerevisiae and C. albicans were inoculated into the YPGal medium and incubated with continuous shaking at 30°C for 9.5 and 8 h, respectively. Cultured cells were harvested and washed thrice with distilled water. The pellets were frozen in liquid nitrogen and stored at −80°C until isolation of mitochondrial proteins was performed. Mitochondrial proteins were isolated from cultured cells following the manufacturer’s instructions and suspended in 10 mM potassium phosphate buffer (pH 7.4). The mitochondrial solution was repeatedly freeze-thawed thrice in liquid nitrogen just prior to measurement. Protein concentrations were determined using the DC protein assay following the manufacturer’s instructions. The enzymatic activities of yeast respiratory chain complexes were measured using a spectrophotometric assay as previously described (17, 37). An appropriate amount of mitochondrial protein was added to assay buffer to yield the same reaction rates (100 mAbs [absorbance]/min). In S. cerevisiae, 20, 40, and 20 μg of mitochondrial proteins were added to assay buffer to measure the activities of complex II, complexes II plus III, and complex IV, respectively. In C. albicans, 60, 7 to 9, 12, and 12 to 15 μg of mitochondrial proteins were added to assay buffer to measure the activities of NADH dehydrogenase, complex II, complexes II plus III, and complex IV, respectively. All measurements were conducted at room temperature. In these experiments, 10 μM rotenone, 10 mM malonic acid, 1 mM KCN, and both 10 mM malonic acid and 10 μg/ml antimycin A served as positive controls of the inhibition of NADH dehydrogenase, complex II, complex IV, and complexes II plus III, respectively (17, 37). In all measurements, agents were added just prior to initiation of the reaction.

The enzymatic activities of bovine heart mitochondrial respiratory chain complexes were determined using MitoCheck complex I, complex II/III, and complex IV activity assay kits (Cayman Chemical, Ann Arbor, MI, USA). Bovine heart mitochondria from these kits were used for experiments. The experiments were performed following the manufacturer’s instructions. In the experiments, 2 μM rotenone, 1 mM KCN, and both 1 mM TTFA and 5 μg/ml antimycin A served as positive controls for the inhibition of complex I, complex IV, and complexes II plus III, respectively.

In all experiments, the reaction rate of each enzyme was determined by calculating the slope of the linear portion of the curve. Complex I activity in C. albicans was calculated by subtracting the rotenone-insensitive enzymatic activity (with 10 μM rotenone) from the NADH dehydrogenase activity (without rotenone).

### Measurement of intracellular ATP levels.

Intracellular ATP levels were measured using a previously described method with the following modifications (25). S. cerevisiae was grown overnight in a semisynthetic medium (per liter: 20 g of lactate, 0.5 g of glucose, 3 g of yeast extract, 1 g of KH_2_PO_4_, 1 g of NH_4_Cl, 0.5 g of CaCl_2_·2H_2_O, 0.5 g of NaCl, and 0.6 g of MgCl_2_·6H_2_O; adjusted to pH 5.5 with NaOH). The cells were harvested during the logarithmic-growth phase by centrifugation (3,000 × *g* at room temperature for 5 min). They were then washed and resuspended in distilled water. The suspension was diluted in a semisynthetic medium to yield 1 × 10^6^ cells/ml. After 5 min of incubation at 30°C, T-2307 and antimycin A were added to the suspension. Further, the cells were incubated for 8 h at 30°C. Subsequently, the cells were collected by centrifugation (4,200 × *g* at 4°C for 5 min). After washes performed with phosphate-buffered saline (PBS; pH 7.2), the cells were resuspended in PBS and diluted to yield 1 × 10^6^ cells/ml. ATP levels were measured using the BacTiter-Glo microbial cell viability assay (Promega, Madison, WI, USA), following the manufacturer’s instructions. Luminescence was measured using a microplate reader POLARstar Optima (BMG Labtech GmbH). To determine the viable cell counts, the cell suspension was diluted with PBS and spread on YPD agar. After a 2-day incubation at 30°C, colonies were counted. The intracellular ATP level is expressed as relative light units (RLU)/CFU.

### MIC determination.

MIC was determined using the broth microdilution method based on the Clinical and Laboratory Standards Institute method with the following minor modifications (38). The medium used for MIC determination was semisynthetic medium, which was used for the measurement of intracellular ATP levels as described above. The final inoculum concentration was 1.5 × 10^3^ cells/ml. The microplate was incubated for 3 days at 30°C. MIC was defined as the lowest concentration at which a score of 2 (approximately 50% inhibition) was observed.

### Statistical analysis.

OCRs in isolated mitochondria and intracellular ATP levels were compared between the control and treatment groups using the parametric Dunnett’s multiple-comparison test. *P* values of <0.05 (comparison of multiple groups) were considered statistically significant. IC_50_ values were determined from dose-response curves compared with untreated control samples using a four-parameter logistic curve-fitting analysis. Data were analyzed using JMP 13.0 (SAS Institute Japan Ltd., Tokyo, Japan).

## Supplementary Material

Supplemental file 1
