# Supplementary material for: The Novel Arylamidine T-2307 Selectively Disrupts Yeast Mitochondrial Function by Inhibiting Respiratory Chain Complexes
Source: Antimicrob Agents Chemother. 2019 Jul 25;63(8):e00374-19. doi: 10.1128/AAC.00374-19 (PMC6658782; doi:10.1128/AAC.00374-19)
Supplement: Supplemental file 1 [file AAC.00374-19-s0001.pdf]

Table S1 Percent inhibition values of positive controls against respiratory chain complexes in *Saccharomyces cerevisiae, Candida albicans*, and bovine heart mitochondria

**Source**	**Positive control**	**Inhibition (%)**		
		S. cerevisiae	***C. albicans***	**Bovine heart**
NADH dehydrogenase (including Complex I)	Rotenone	Non-existence (complex I)	59	68
Complex II	Malonic acid	100	100	Not determined
Complex II+III	Malonic acid	97	98	Not determined
	TTFA	Not determined	Not determined	86
	Antimycin A	97	98	92
Complex IV	KCN	99	100	99

The values represent the mean values of pooled data from two independent experiments, each performed in triplicates in *S. cerevisiae* and *C. albicans* mitochondria and 6–8 times in bovine heart mitochondria except KCN. The value of KCN in bovine heart mitochondria represents the mean value of pooled data from two independent experiments, each performed in triplicates.
